# Geostatistical and multi-criteria decision-making models to subsidize a quantitative monitoring network of aquifers intensively used for irrigated agriculture: the case of Urucuia Aquifer System, Brazil

**DOI:** 10.1007/s10661-026-15324-y

**Published:** 2026-04-29

**Authors:** Bernardo Ramos Carneiro Leão, Gerson Cardoso da Silva Junior, Eduardo Antonio Gomes Marques

**Affiliations:** 1 Department of Geology, Federal University of Rio de Janeiro – UFRJ, Ed. CCMN Bloco J Sala J0-05 - Av. Athos da Silveira Ramos 274, Rio de Janeiro, RJ 21941-916 Brazil; 2 Civil Engineering Department, Federal University of Viçosa – UFV, Av. P H Rolfs, S/N, Campus Universitário, Viçosa, MG 36570-000 Brazil

**Keywords:** Sedimentary aquifers, Bahia State, Brazil, Groundwater management, Monitoring network design, Urucuia Aquifer System

## Abstract

Groundwater monitoring is essential for effective water resource management, enhancing hydrogeological understanding and supporting public policy implementation. In Brazil, systematic groundwater level monitoring started only in the 2010s, with the Brazilian Geological Survey (SGB) “Integrated Groundwater Monitoring Network” (RIMAS). Among Brazilian aquifers, the Urucuia Aquifer System (UAS) is the most monitored, covering 126,468 km^2^, primarily in the State of Bahia, northeastern Brazil. However, with a density of just 0.0008 wells/km^2^, monitoring remains incipient as compared to international standards. The UAS, a predominantly phreatic aquifer composed by fluvial-eolian Cretaceous sandstones from the Urucuia Group, Sanfranciscan Geological Basin, plays a strategic role in economic and social development and sustains baseflows in the São Francisco River basin. Rapid agricultural expansion in western Bahia, with increasing groundwater withdrawals, required improved management through a functional monitoring network. This study developed a multi-criteria model to identify priority areas for expanding the UAS monitoring network. It considered hydrogeological, physiographic, water abstraction, and well availability factors, integrated with geostatistical analysis. Results confirmed the shortages of the current network. Vulnerable areas identified include the western Corrente River basin and the Branco, Rio de Janeiro, and Ondas River sub-basins, which should be prioritized for network expansion. The study highlights the effectiveness of integrated methods for designing monitoring networks, a relatively unexplored approach in Brazil. These findings contribute to improve the decision-making process and a deeper understanding of the UAS hydrogeological context, paving the way for better management of groundwater resources in the region.

**Supplementary Information:**

The online version contains supplementary material available at 10.1007/s10661-026-15324-y.

## Introduction

Groundwater is a paramount component of the hydrological cycle, constituting large freshwater reservoirs, storing and distributing water on large spatial and temporal scales through subsurface flow. Thus, groundwater monitoring is imperative for good water resource management, improving long-term understanding of the hydrogeological system by systematically collecting quantitative and qualitative data (Condon et al., 2021; Hosseini & Kerachian, 2017; Neupane et al., 2014).

In Brazil, the creation of a groundwater monitoring policy is very recent, with a still incipient national groundwater monitoring network, initially implemented in the early 2010s. The Integrated Groundwater Monitoring Network (RIMAS) project, created in 2009 by the Geological Survey of Brazil (SGB), took into account basically hydrogeological aspects in the allocation of the monitoring points (Mourão & Peixinho, 2012; SGB, 2009).

The Urucuia Aquifer System (UAS) is the most intensively monitored aquifer in the RIMAS context. The UAS is a regional groundwater reservoir with an area of 126,468 km^2^ covering the states of Goiás, Maranhão, Minas Gerais, Piauí, Tocantins, and Bahia, the latter containing around 65% of the total UAS domain (Gaspar, 2006; Gaspar & Campos, 2007; ANA 2017). The RIMAS network has 67 wells in the western Bahia portion of the aquifer. Even so, due to its large extent, it has a very low density of only 0.0008 wells/km^2^.

The UAS is a predominantly unconfined aquifer, composed of fluvial-eolian sandstones of (bottom to top) Posse and Serra das Araras formations from the Urucuia Group, a Neocretaceous unit belonging to the Sanfranciscan Basin, the Phanerozoic cover of the São Francisco Craton (Barbosa, 2016; Campos & Dardenne, 1997a, 1997b; Gaspar, 2006).

The western portion of Bahia State became an important agricultural frontier in Brazil since the 1980 s, with strong economic development due to the large-scale growing of crop commodities (Costa et al., 2021). This has led to an increase in demand for water resources, with the concession of surface and groundwater permits for agricultural use, intensifying conflicts between the agribusiness, environmental organizations and small traditional rural communities (Carvalho, 2022). In addition to its groundwater potential, with individual wells exceeding flows of 500 m^3^/h, the UAS makes a significant contribution to maintaining the region’s river baseflows, providing up to 95% of the total flow through groundwater discharge, especially during the dry season (Gonçalves et al., 2018; Schuster et al., 2002). Consequently, the aquifer provides up to 36% of the São Francisco River (SFR) annual flow, through the left margin tributaries (Vieira, 2021). The SFR is one of the most important river basins of Brazil, hydraulically and economically. Therefore, the UAS is of strategic importance to the development of the western Bahia region, requiring good management of water resources in order to avoid aquifer overexploitation due to pressures already under course, with the installation of irrigation pivots, and also climate change impacts.

In recent decades, several methodologies have been applied to design quantitative monitoring networks. The main approaches used involve geostatistics to allow an evaluation of existing networks through interpolation by kriging, visualizing areas with redundancy or insufficient monitoring through the average error, as well as optimizing the networks (Bhat et al., 2015; Theodossiou & Latinopoulos, 2006; Triki et al., 2013; Yang et al., 2008). Another approach is the entropy-based methods, which similarly allow the optimization of networks through uncertainty (Leach et al., 2016; Mondal & Singh, 2012). Also, multi-criteria decision-making (MCDM) methods using geoinformatics are another possibility, with the overlay of environmental parameters to define priority zones for monitoring (Esquivel et al., 2015; Kim, 2010). There is also a hybrid approach, with the combination of more than one methodology in order to provide greater consistency in the results (Uddameri & Andruss, 2014; Chandan & Yashwant, 2017, Singh & Katpatal, 2017).

Singh and Katpatal (2020) reviewed and compared quantitative network design methodologies of several studies. The authors concluded that hybrid approaches are the most recommended for establishing an effective groundwater monitoring network, and that it is necessary to consider anthropogenic aspects and the connection with surface water, not just hydrogeological aspects. As described, there are some studies with a hybrid approach (Uddameri & Andruss, 2014; Chandan & Yashwant, 2017; Singh & Katpatal, 2017). However, none of them takes into account aspects related to surface water, which, in the area of this study, has a high connectivity with groundwater, as well as the land use in a context of agricultural expansion.

The aim of the present study is, therefore, to identify priority zones for the implementation of a regional quantitative groundwater monitoring network to complement the RIMAS network in the western Bahia UAS domain, using GIS tools and the geostatistical kriging interpolation. This allowed the evaluation of the existing monitoring network in the study area and identification of areas with maximum error variances, in conjunction with water level data and statistics, as well as application of the MCDM analysis. At the end of the process, we elaborated a model of priority zones for groundwater level monitoring considering integrated surface-water and groundwater aspects, as well as factors related to land use, land cover, and pressure for water use.

## Material and methods

### Study area

**Fig. 1:**
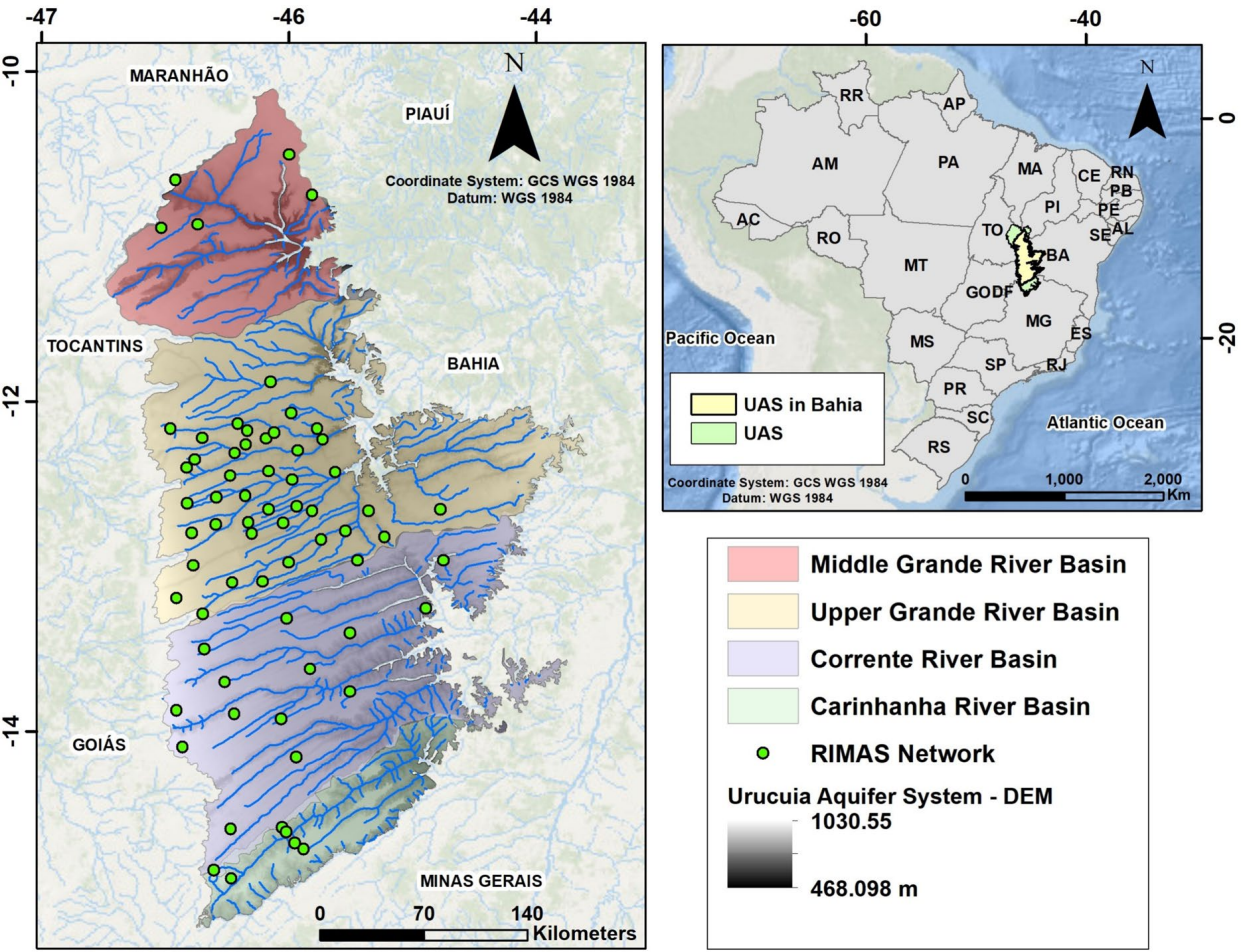
Location map of the study area. The Upper Grande River basin and the Corrente River basin are represented in pale yellow and lilac, respectively. Middle Grande River (also known as Preto River) basin, in red, and Carinhanha River basin, in green, are the other catchments within the region. The 67 RIMAS monitoring wells in western Bahia are shown as green dots

For the present study, the Upper Grande River and Corrente River basins were selected within the domain of the UAS in western Bahia (Fig. 1). This choice was conditioned by the expansion of the agricultural frontier, more significant in those basins. Several sub-basins of the Upper Grande River and Corrente River basins have been considered in previous studies as overexploited (Mantovani et al., 2019).

The region’s climate is characterized by well-defined seasons, with a rainy summer between October and April and a dry winter from May to September, when rainfall is almost absent. Average temperatures range from 26 to 20 °C, while relative humidity fluctuates between 50% in August and 80% in December. Annual rainfall, which has presented a decreasing trend in recent decades, varies from 900 mm in the far east to 1500 mm in the west of the study area (Gaspar, 2006; Mantovani et al., 2019; Pousa et al., 2019).

This spatial and seasonal variability has influenced land occupation, with irrigated crops predominating in the central and eastern portions of the area, and rainfed crops in the west. Since the 1980 s, agricultural expansion has resulted in the suppression of the Cerrado (Brazilian Savannah) native vegetation, which has been replaced by croplands and pasture. Between 1990 and 2020, there was an increase of 3.17 Mha in agricultural areas and 193,480 ha in irrigated agricultural areas (Pimenta et al., 2021). This expansion has increased the demand for water resources, causing a greater use of groundwater, as rivers have reached their maximum limit for water permits from regulatory agencies (Gaspar, 2006; Gaspar & Campos, 2007).

The regional topography is dominated by a plateau with an average 5° slope from west to east, covered by high-infiltration latosols and quartz neosols, which explain the low density of rivers in the region (Campos, 1996). Regarding the hydrography, the UAS contributes significantly to river baseflows, averaging 80–95% of the total runoff, thus highlighting its ecological and socio-economic importance (Gonçalves et al., 2018; Vieira, 2021). The drainage pattern of the rivers has strong structural control, showing a parallel to subparallel NE-E drainage pattern conditioned by the reactivation of existing structures in the Bambuí Group unit from São Francisco Craton (Campos, 1996), and tributaries with a rectangular pattern caused by secondary fracturing, in an approximately NW-SE direction (Pereira, 2023).

The study area is located in the Sanfranciscan Basin, which covers approximately 160,000 km^2^ in the São Francisco Craton (Campos & Dardenne, 1997b; ANA, 2017). With an elongated north-south format, the basin is limited by the Parnaíba Basin in the north and the Paraná Basin in the south. To the east, it is bordered by the Araçuaí/Espinhaço Setentrional Belt, while to the west, it is bordered by the Brasília Belt (Batezelli & Ladeira, 2016; Campos & Dardenne, 1997b). The basin sediments are laid in erosive discordance over various formations of the basement, most markedly and representatively by rocks of the Bambuí Group (Campos & Dardenne, 1997a; Carvalho, 2022).

The Urucuia Group, of Neocretaceous age sedimentation, makes up the UAS, including the Posse and Serra das Araras Formations, domaining units in the study area (Barbosa et al., 2017). The Posse Formation (basal) is formed by fine to medium-grained sandstones with cross-stratification and high compositional and textural maturity, associated with aeolian and fluvial systems. The upper Serra das Araras Formation has medium to thick-grained sandstones with conglomeratic lenses, discontinuous silicification, and oxidizable pelitic levels, interpreted as fluvial deposits in lowlands, with flow variation (Campos & Dardenne, 1997a; Gaspar, 2006; Mantovani et al., 2019).

The Urucuia Aquifer System is a group of aquifers within the Urucuia Group. It is predominantly unconfined, intergranular, with regional extension. The hydrogeological basin is asymmetrical, with a N-S longitudinal axis that separates the groundwater flow to the basins of the Tocantins-Araguaia River to the west and the São Francisco River to the east (Gaspar & Campos, 2007; Barbosa, 2016; ANA, 2017; Carvalho et al., 2023).

**Fig. 2:**
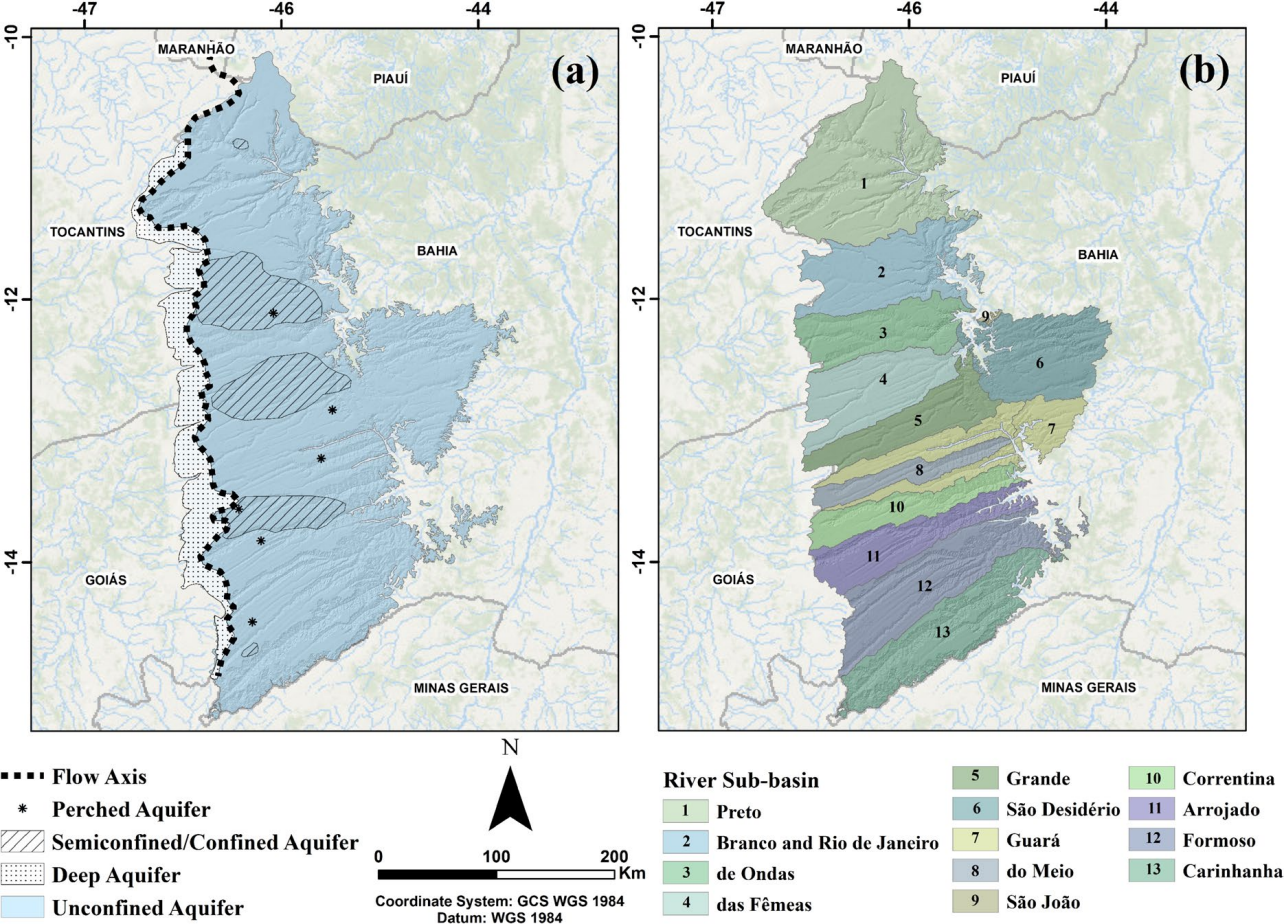
**a** Map showing the subtypes of aquifers present in the Urucuia Aquifer System portion, located in the State of Bahia (UAS-BA), modified from Gaspar and Campos (2007). **b** Sub-basins present in the UAS-BA domain

Although there is still ongoing research into the existing aquifers and their interrelationships, Gaspar and Campos (2007) proposed four subtypes of aquifers (Fig. 2a): Regional Phreatic Aquifer, with greater spatial relevance; Local Perched Aquifer, formed by levels of silicified sandstones at the base of Serra das Araras Formation, which trap water above the regional potentiometric level; Confined/Semiconfined Aquifer, also controlled by levels of silicified sandstones, showing slow drainage and artesian conditions, locally important in areas such as the city of Luís Eduardo Magalhães, in the central portion of the study area; and Deep Unconfined Aquifer, present in the extreme west, close to the regional flow dividing axis, reaching depths of over 100 m, with a high hydraulic gradient.

The wells in the region reach flow rates up to 500 m^3^/h and specific capacity between 17.2 and 23 m^3^/h/m, with small drawdowns (Barbosa, 2016; Gaspar & Campos, 2007; Schuster et al., 2002). ANA (2017) and Mantovani et al. (2021) suggest exploitable reserves of between 3.72 and 4.84 km^3^/year, with up to 18% already used in the Grande River basin. Both studies consider a sustainability coefficient (Cs) of 0.2 to balance the exploitation of the aquifer with the maintenance of river flows, meaning that only 20% of the exploitable reserves should be granted.

### Statistical and hydrogeological analysis

Statistical analysis was carried out to characterize the dispersion of the groundwater level (GWL) data from 58 RIMAS wells in the Upper Grande River and Corrente River basins throughout the monitoring period analyzed in this study, from 2011 to 2023. Although the focus of the work is on the above-mentioned basins, where agricultural expansion is more important, 10 wells distributed in the Middle Grande River and Carinhanha River basins were also selected for this analysis, to compare the dispersion data with the results obtained in the target basins.

To measure dispersion, the variance values were calculated, which are measures of dispersion that show the distance between each value in a set and the central (average) value, and the standard deviation values, which analyze the distance from the mean of a group of numbers, using the square root of the variance.

After obtaining the results of this basic statistical analysis, interpretation was performed in combination with the hydrogeological features of the study area, considering the flow divisor of the hydrogeological basin and granted groundwater extraction wells.

### Geostatistical analysis

Hydraulic head data were collected from 58 RIMAS monitoring wells between 2011 and 2023, in the area of interest and processed in a GIS environment using the Geostatistical Analyst tool in ArcGIS 10.8.2 software. Hydraulic head was the unit of measurement used in this analysis, because it is a scalar quantity that indicates the direction of groundwater flow across the potentiometric surface, with different values measured in the wells, since the same reference datum is used, in this case sea level. This avoids possible misinterpretations due to local topographic variations from where the wells are located. Geostatistics is a class of statistical methods used to analyze and predict values associated with geographical location and spatial dependence (Caers, 2005; ESRI, 2013). The spatial variability of a regional variable is described by the semivariogram, represented in Eq. 1:

1 $$\gamma \left(h\right)= \frac{1}{2n\left(h\right)}\sum_{i=1}^{n\left(h\right)}{[(z\left(x+h\right)-z\left(x\right))}^{2}]$$

where the semivariogram is the graphical representation of the mean square variability between two neighboring points at a distance $h$, with $z(x)$ and $z(x\mp h)$ the values of the variable at point $x$ and at a point $h,$ away from point $x$.

Ordinary kriging is the geostatistical interpolation method in which the average of the regionalized variable is assumed to be constant throughout the area of interest. The general kriging equation is represented in Eq. 2:

2 $$Z\left({x}_{0}\right)=\sum_{i=1}^{n}{\lambda }_{i}Z({x}_{i})$$

where $Z\left({x}_{0}\right)$ is the value to be calculated at location ${x}_{0}$; $Z({x}_{i})$ is a known value at location ${x}_{i}$; ${\lambda }_{i}$ is the weight calculated based on the distance, the semivariogram, and the spatial relationships of the measured values along the predicted locations; and $n$ is the number of samples.

The difference between the estimated and measured values tends to be zero, in order to make the prediction unbiased. To ensure that the prediction is unbiased, the sum of the weight ${\lambda }_{i}$ must be equal to 1 and Eq. 3 must be solved simultaneously to minimize the limitation:

3 $$\sum_{i=1}^n\lambda_i=1;\overset n{\underset{i=1}{\;\sum}}\lambda_i\gamma\left(x_i,x_j\right)-\mu=\gamma(x_i,x)$$

where $\mu$ is Lagrange’s multiplier; $\gamma \left({x}_{i},{x}_{j}\right)$ are the estimated values of the semivariogram based on the distances between the samples observed at the locations ${x}_{i}$ e ${x}_{j}$; and $\gamma ({x}_{i},x)$ are the estimated values of the semivariogram based on the distances between the point ${x}_{i}$ and the expected location $x$.

Therefore, to apply ordinary kriging interpolation, the data must have stationary behavior, in which the mean value of the expected variation within a neighborhood area is constant, i.e., without a trend. It is therefore necessary to remove the data trend, as a function of the coordinates, by adjusting a polynomial equation of order “n” using the least squares method, in which the residuals will later be used to adjust the semivariogram of the variable under study. To identify the trend in the data more precisely, graphs were drawn up representing the hydraulic head values in relation to the spatial distribution of the monitoring wells and considering the first, second, and third order polynomial regression adjustments.

Through kriging, it is possible to estimate the values of the regionalized variable where there are no initial measurements by interpolating the error. It results in the interpolation error increasing with the uncertainty of the prediction, due to the density of the monitoring network. This indicates possible areas for the allocation of new monitoring points (Theodossiou & Latinopoulos, 2006; Chandan & Yashwant, 2017).

In addition, cross-validation was carried out to assess the relative importance of each existing point in the RIMAS network, along with the use of critical sense, with reflections on the hydrogeology of the region, considering that it is an aquifer system, with different potentiometric surfaces. Cross-validation is based on carrying out consecutive interpolations with the removal of one monitoring point at a time, to assess the differences between the measured and estimated water level values. The statistical analysis of the water level variability in the monitoring wells over time was also carried out, measuring the standard deviation and variance to identify the most important wells in the network, in comparison with data from water grants in the region and neighboring basins.

### MCDM parameters and steps

The multi-criteria analysis method used in this study consists of collecting data on land use and land cover (LULC) from the multi-disciplinary network MapBiomas (https://mapbiomas.org/), water level variation (m), rate of water level decline (m/year) from the RIMAS network wells, measured from 2011 to 2023, and density of granted interference points obtained from the Bahia State Environmental Agency (INEMA-BA). The maps were generated and processed in the ArcGIS 10.8.2 environment. Afterwards, they were superimposed using map algebra, generating a final map with priority zones to be monitored.

#### Standardization of parameters

After producing the parameter raster maps, we normalized the parameters to be considered, since they have different scales and units. The fuzzy parameter integration function was used, making it quick and feasible to normalize the criteria on a scale ranging from 0 to 1 (Eastman, 2012; Honghai & Altinakar, 2013; Chandan & Yashwant, 2017). The normalization formula is represented by Eq. 4:

4 $${X}_{i}=\frac{{(R}_{i}-{R}_{min)}}{{(R}_{max}-{R}_{min})}$$

where ${X}_{i}$ is the normalized value, ${R}_{i}$ is the original parameter value, ${R}_{\mathrm{min}}$ is the lowest value of the parameter, and ${R}_{max}$ is the highest value of the parameter.

#### Weighting using Analytical Hierarchy Process (AHP)

Once the criteria had been normalized, the relative weighting of these factors was carried out using the Analytical Hierarchy Process (AHP), developed by Saaty (1977), by constructing matrices comparing the parameters with each other in an order of priority ranging from 1 (least important) to 9 (most important). Within the matrix, the parameters were compared according to the columns and rows, assigning integer values or fractions depending on their relative importance (Sener et al., 2010; Sener & Davraz, 2013; Jenifer & Jha, 2017).

In this work, the following order of hierarchy was defined, from the most important criterion to the least important: (1) density of interference points, (2) rate of decline in water level, (3) variation in water level, and (4) land use and occupation.

This order of hierarchy was defined in such a way that the density of granted points of interference indicates locations that imply a higher stress on the region’s water resources, due to the greater number of wells and surface water abstractions with high extraction flows.

Next, we considered the rate of decline in the water level, a direct measurement that represents possible areas where the aquifer is being most affected. Also, the water level variation represents, in general, how the hydrodynamics of the aquifer responded to extraction and climate effects during the monitored period.

Finally, the LULC indicates areas where agricultural expansion has been more relevant. It was defined as a less important criterion because, even if land used for agriculture lowers the infiltration capacity of water in the soils, this impact is not so significant to recharge, since the infiltration capacity remains relatively high, as demonstrated by Eger et al. (2021).

After weighting, matrix calculations are carried out to define parameters and calculate the Consistency Index (CI), obtained according to Eq. 5:

5 $$\mathrm{CI}=\frac{{\lambda }_{\mathrm{max}}-n}{n-1}$$

where ${\lambda }_{\mathrm{max}}$ is the average of $\lambda (\mathrm{eigenvalue})$ values, resulting from the vector product, divided by the eigenvector (weight of each criterion) of the matrix, and $n$ is the order of the matrix.

The consistency ratio (CR) can then also be calculated, which, if it results in a value less than or equal to 0.1 (CR ≤ 0.1), indicates that the weighting has been carried out properly (Saaty, 2003). The calculation of the consistency ratio is shown in Eq. 6:

6 $$\mathrm{CR}=\frac{{\lambda }_{\mathrm{max}}-n}{\left(n-1\right)\times \mathrm{RI}}$$

where $\frac{{\lambda }_{\mathrm{max}}-n}{\left(n-1\right)}$ is the Consistency Index (CI), and $\mathrm{RI}$ is the Random Index, which is obtained as a function of the order of the matrix.

### Overlapping criteria

In the final stage, after calculating the CR and CI to validate the weighting of the parameters, they are overlaid in GIS using raster calculator and map algebra, multiplying the factors by their associated weights (eigenvectors), and Boolean maps that have already been standardized (Eastman, 2012). After overlapping, the final multi-criteria model is generated, which can vary from 0 to 1 in priority areas for monitoring. The weighted overlap of the criteria can be explained by Eq. 7 (modified from Marinoni (2004)):

7 $$\mathrm{ZP}= \sum_{\mathrm{i}=1}^{\mathrm{n}}{W}_{\mathrm{i}} {X}_{\mathrm{i}}$$

where $\mathrm{ZP}$ are the priority zones for monitoring, $n$ is the number of parameters, ${W}_{\mathrm{i}}$ is the relative weight $i,$ and ${X}_{\mathrm{i}}$ is the value of *i*.

## Results and discussion

### Data dispersion and hydrogeological interpretation

**Fig. 3:**
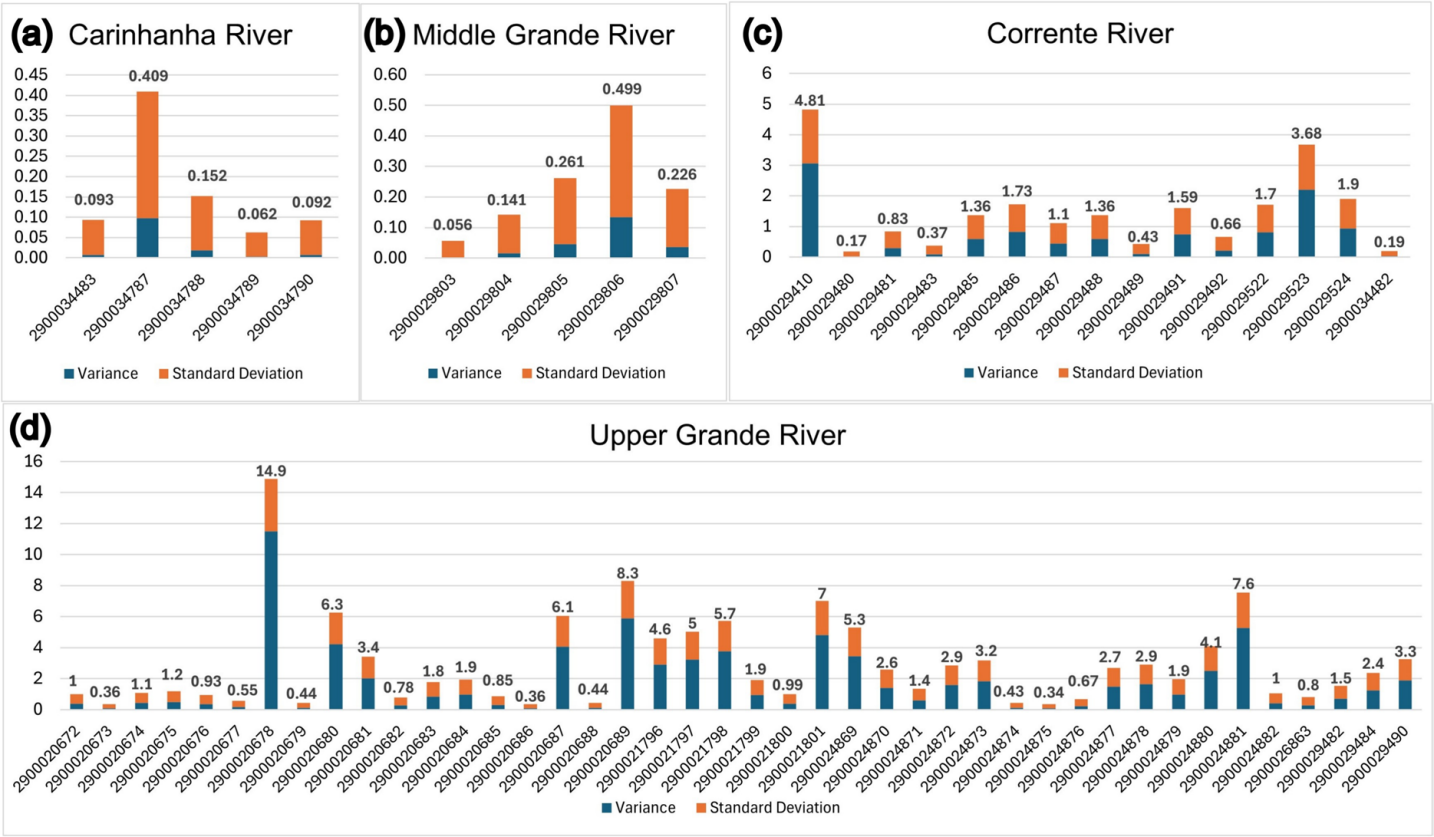
Variance (navy blue) and standard deviation (orange) values for the Carinhanha (**a**), Middle Grande (**b**), Corrente (**c**), and Upper Grande (**d**) river basins. The *y*-axis represents the dispersion values, while the *x*-axis represents the wells’ identification. The sum of the calculated parameters is shown at the top of the bar for each monitoring well

Before applying the geostatistical method with ordinary kriging interpolation, a statistical analysis was carried out, where 43 wells were analyzed in the Upper Grande River basin, with variances ranging from 0.08 to 11.48, and standard deviations ranging from 0.28 to 3.39 (Fig. 3d). In the Corrente River basin, the calculated variance values ranged from 0.02 to 3.06, and standard deviations ranged from 0.15 to 1.75 (Fig. 3c) in the 15 wells analyzed.

The same analysis was carried out for the 10 wells in the Middle Grande River (Fig. 3b) and Carinhanha River (Fig. 3a) basins. The analysis of the graphs shows that the dispersion in the GWL data for the last-mentioned basins is much smaller than the basins that are the focus of this study, with the sum of the variance and standard deviation values not reaching 0.5 for the Middle Grande River basin and 0.45 for the Carinhanha River basin.

**Fig. 4:**
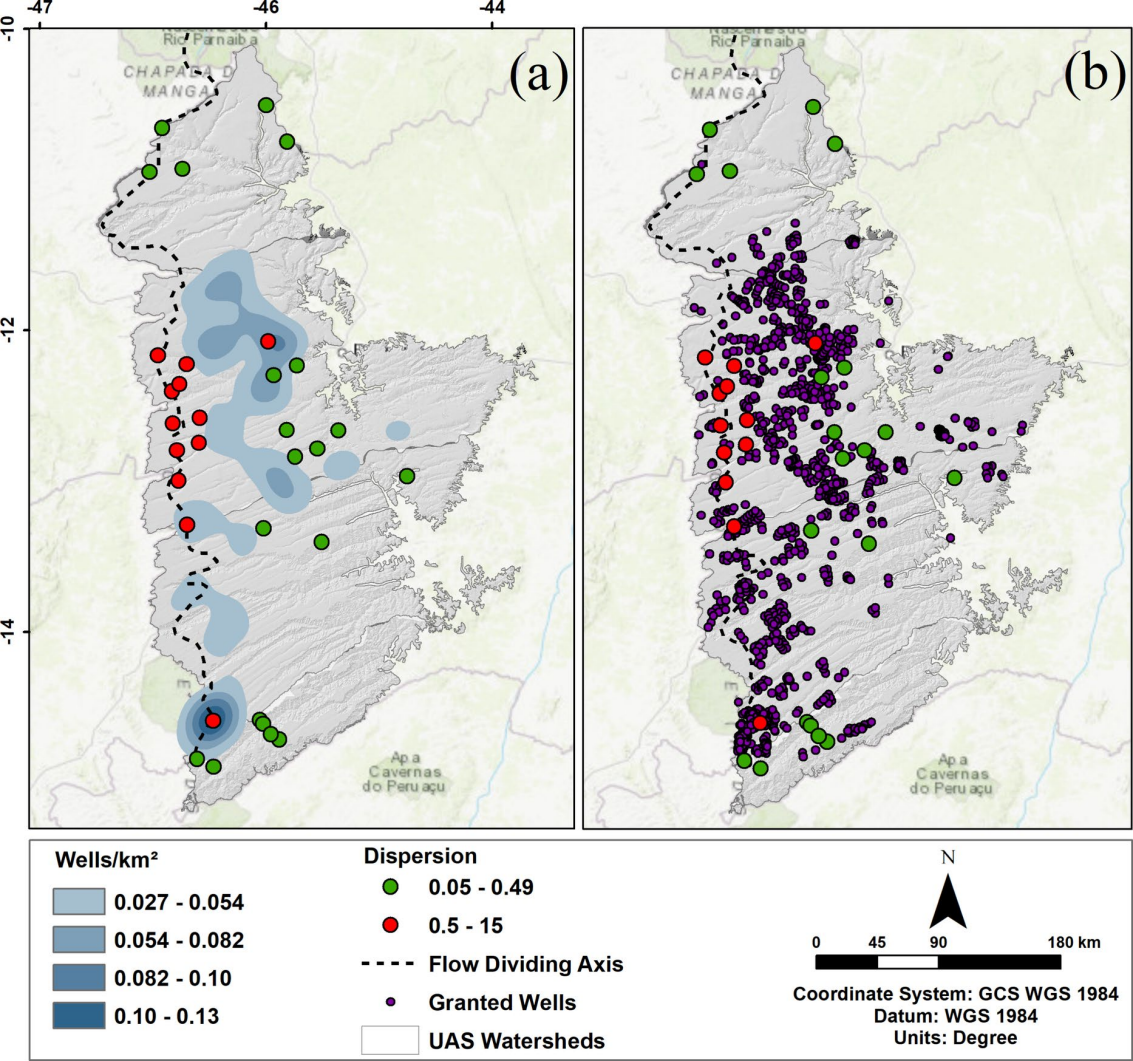
RIMAS wells with the higher (red dots > 0.5) and lower (green dots < 0.5) dispersion values, compared with INEMA’s granted wells (**a**), and areas with the higher densities of granted extraction wells (**b**)

The higher dispersion values occur in wells positioned close to the limit of the watershed in the extreme west of the study area, as shown in Fig. 4. This is because the groundwater table near the flow dividing axis suffers more abrupt variations as storage changes in the aquifer draining east to the São Francisco River and west to springs on the Serra Geral de Goiás escarpment towards the Tocantins River. The wells with higher dispersion tend to present greater ranges in GWL values, measured over the monitoring period. Although there are wells in the Middle Grande and Carinhanha basins with low dispersion, and close to the flow dividing axis, it is important to point out that the axis defined by Gaspar (2006) and Gaspar and Campos (2007) is inferred in these areas, in addition to the fact that these basins are still comparatively underexploited.

Although the number of wells in the Carinhanha and Middle Grande river basins is considerably smaller, the analysis remains valid, since out of the 58 wells that are included in the Upper Grande and Corrente watersheds, only 10 (4 in the Corrente and 6 in the Upper Grande), which are equivalent to approximately 17% of the sample, have dispersion values lower than the highest dispersion value observed in the Carinhanha and Middle Grande data set. This emphasizes that the standard deviation and variance values are overall higher in the Upper Grande and Corrente basins.

The larger amount of pumping data in the Upper Grande and Corrente basins may respond for the higher variance and standard deviation values in these basins compared to the Middle Grande and Carinhanha basins. These basins are much less stressed by groundwater and surface water extraction. This is corroborated by Fig. 4a, where wells No. 2900020678 and 2900021796 (isolated red dots in the southwest and middle east areas, respectively) are inserted in locations with the higher density of licensed extraction wells. The last well mentioned is the only one in the group with the higher dispersion that is not located in the western region of the study area.

As for the wells with lower dispersion (values less than 0.5), of the 20 points selected, 10 are in the Middle Grande and Carinhanha basins. The remaining wells are located in areas less burdened by water resource exploitation in the central-eastern region of the study area, with three of them in the Corrente River basin, and six in the Upper Grande River basin.

### Standard prediction error interpolation

Potentiometry data in the region tends to follow the topographic gradient, so it was possible to notice a 1st-order trend with a better fit to the hydraulic head data. This effect was removed in the process of modeling the semivariogram and interpolation by ordinary kriging. After removing the first-order trend surface, a variographic modeling of the residues was carried out, where different types of semivariograms were tested and the best fit was obtained with the spherical model.

**Fig. 5:**
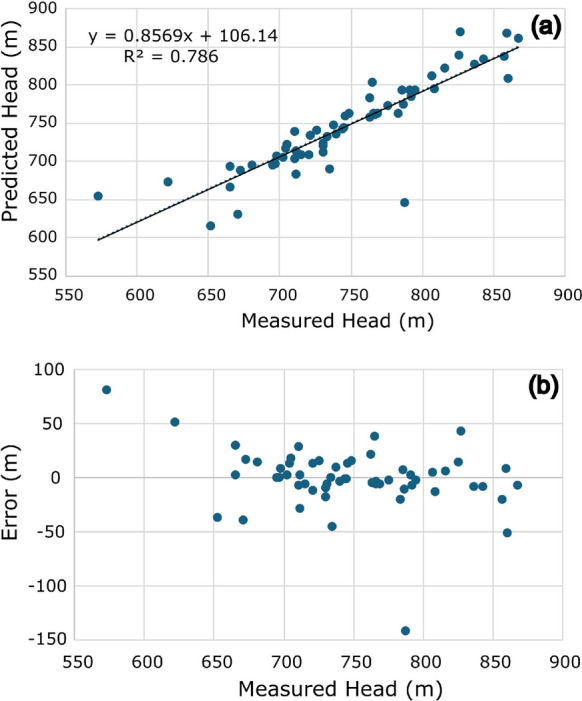
**a** Cross-validation linear regression showing the correlation between estimated data (*y*-axis) and measured data (*x*-axis). **b** Comparative graph showing the correlation between the measure error (*y*-axis) and the measured data (*x*-axis)

**Fig. 6:**
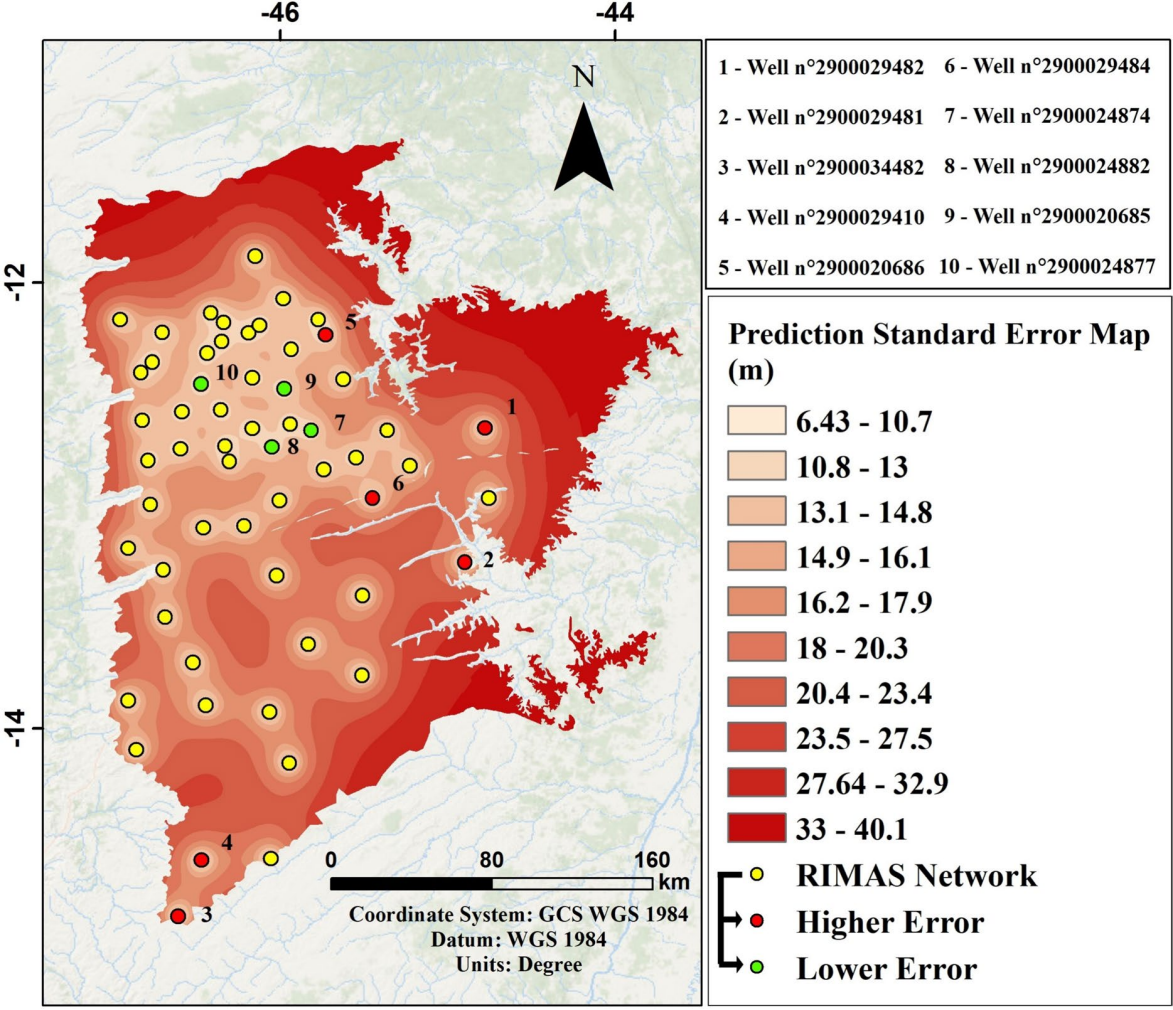
Illustrative map of the prediction standard error surface in the Upper Grande and Corrente river basins, defined using ordinary kriging interpolation. The locations with the lower density of monitoring points have the higher associated errors

Once the semivariogram was established, it became possible to cross-validate the data and find the difference between the measured and estimated values through interpolation (Fig. 5a). Additionally, it allowed the determination of the values (Fig. 5b) and the surface of the prediction standard error of the hydraulic head measurements along the two basins of interest (Fig. 6).

The linear regression graph (Fig. 5a) between the estimated and measured data showed a coefficient of determination (*R*^2^) of 0.786. The further the points are from the line, and therefore from the measured value, the greater the associated error. This allows an assessment of the existing network, defining points of greater relative importance.

Analysis of the measured and estimated values in the cross-validation together with the error shows that 9 of the 58 interpolated points (15.52%) have estimated values with an associated error of more than 30 m in comparison to the measured values, while 18 points (31.03%) are within the 6 m range. This result shows high variance due to the low number of monitoring wells relative to the large area to be monitored. The wells with the higher associated errors are in outlying areas, such as wells 2900029482 and 2900029481, located in the São Desidério and Guará River sub-basins both in the eastern part of the study area, and wells 2900034482 and 2900029410, in the Formoso River sub-basin (see Fig. 2b), in the western part of the study area. The exceptions are wells 2900020686 and 2900029484, located in more densely populated areas of the Upper Grande River basin, but which will be discussed later on in correlation with the hydrogeological data.

The interpolation of the standard error highlighted the fact that, in general, more densely populated sites have lower variance compared to the outlying zones of the study area. The sub-basins of the Ondas River to the north in the area of interest, the São Desidério River to the east, and the Formoso River to the south are the regions with the higher standardized mean errors and are the priorities for installing the complementary network, according to the methodology applied here.

### Parameters weighted overlay

**Fig. 7:**
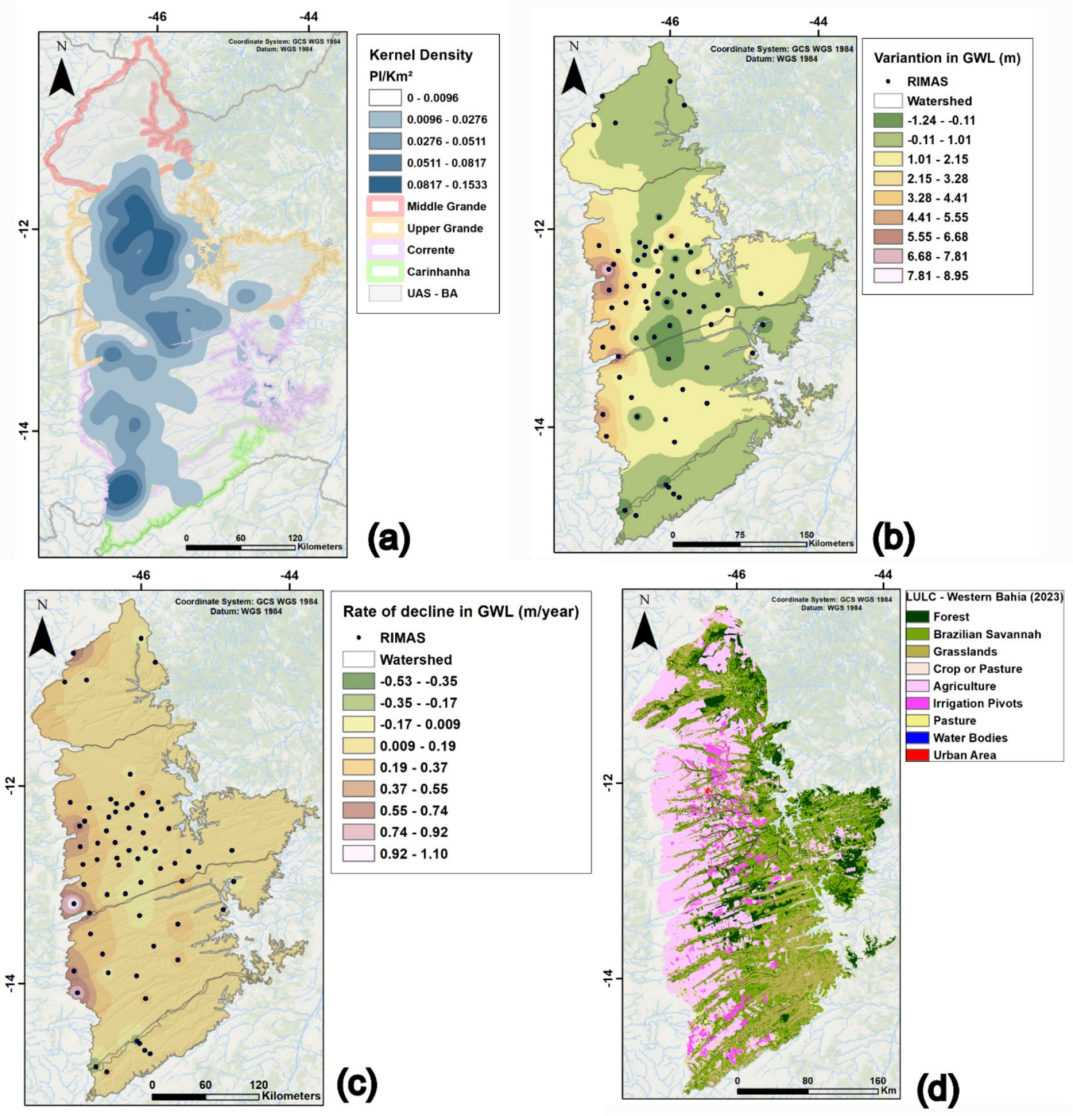
Parameters used in the multi-criteria model. **a** Kernel density for interference points per square kilometer. **b** Water level variation in the UAS domain between 2011 and 2023. **c** Rate of water level decline in the UAS, considering data from the RIMAS wells in the period from 2011 to 2023. **d** LULC for the UAS domain in western Bahia, 2023. Obtained and modified from MapBiomas (2024)

Initially, maps were generated for the parameters to be superimposed in the MCDM analysis, to visualize the data spatially and allow an initial analysis of the influence of each criterion separately in the study area (Fig. 7). Although the methodology was applied to the Upper Grande and Corrente River basins, as in the statistical analysis, the initial parameters were represented throughout the aquifer domain in western Bahia, for a better interpretation of the spatial distribution.

The density map of groundwater and surface water interference points (Fig. 7a) was built using the kernel density function, which calculates the density of point features around each raster cell. In this case, INEMA’s grant data were used, which represent the points that have been regularized, and are authorized to extract surface or groundwater resources.

The analysis of this parameter alone clearly shows that the Upper Grande and Corrente River basins have the highest number of granted interference points in the UAS domain in western Bahia. On a more specific sub-basin scale, the northern portion of the Upper Grande River basin, the Branco, Rio de Janeiro, de Ondas, and das Fêmeas River sub-basins have a larger number of extraction points in this area, while the Formoso River sub-basin tends to have the largest volume of extraction in the Corrente River basin.

The GWL variation map was generated using IDW (inverse distance weighted) interpolation of GWL data from 66 RIMAS monitoring wells for the period between 2011 and 2023. The IDW interpolation method assigns weights to the sample data (wells), where the influence of one point on another increases with proximity and consequently decreases with distance from the new point to be estimated. It is assumed that points that are closely located are more similar than those that are far apart. Figure 7b illustrates the result of applying this method to the entire UAS-BA domain.

The small number of monitoring wells in the Middle Grande River (five wells) and Carinhanha River (four wells) basins is noteworthy, making it difficult to make a better diagnosis of these basins, although the results in general indicate stability of the GWL over the monitored period.

The results of the interpolation of the GWL variation monitored in the RIMAS wells for the two basins of interest show greater drawdown in the far west of the study area, close to the aquifer’s flow divider axis. The accumulated drawdown in this region reaches almost 9 m in the Upper Grande River basin and more than 5 m in the Corrente River basin.

The water level decline rate map (Fig. 7c) was also produced using IDW interpolation. This parameter deals with the rate of annual decline and is derived from the GWL variation map, in which the rate of decline is calculated using the total decline divided by the period monitored. This parameter allows us to see which wells show the greatest relative decline, since the points do not have the same monitoring period, with some starting after 2011. Figure 7c also shows that the central-southern region of the aquifer as a whole shows relative stability of the water level over time, while the western portion shows a decline with rates close to 1 m/year in certain regions, especially at the headwaters of the Ondas, das Fêmeas, and Grande rivers in the Upper Grande River basin, and the Correntina and Arrojado rivers in the Corrente River basin.

The last parameter used was LULC (Fig. 7d), which allowed the observation of the expansion of rainfed and irrigated agriculture in the western region of Bahia in shades of pink and purple on the map, mainly in the area of the Grande River basin. The areas with the higher concentration of irrigation pivots are the sub-basins of the Branco, Rio de Janeiro, and de Ondas rivers in the Upper Grande River basin, and also in the sub-basin of the Formoso River in the Corrente basin, a result similar to that obtained from the kernel density.

The LULC criteria were normalized using an arbitrary hierarchical order, as they are qualitative parameters. With this, arbitrary values were assigned, ranging from 0 to 1 depending on the land use class, considering the impact on the region’s water resources, such as lower infiltration rates and greater water abstraction, as in the case of pivots. The classification of LULC is shown in Table 1. Classes with equal weights were merged into a unified class using the reclassify tool in an ArcGIS environment.

**Table 1:** Normalized weight assigned to LULC through arbitrary hierarchical ordering

*LULC class*	*Weight*
Water bodies	0.001
Brazilian Savannah/grasslands	0.20
Pasture	0.50
Rainfed agriculture	0.70
Irrigated agriculture/urban sreas	1

After normalizing the criteria, the matrices were constructed and weighted using the Analytical Hierarchy Process (AHP), as shown in Table 2.

**Table 2:** Weighted hierarchy matrix with relative importance between the parameters used in the model

*Parameters*	*Density*	*GWL decline rate*	*GWL variation*	*LULC*
*Density*	1	2	3	6
*GWL decline rate*	0.50	1	2	5
*GWL variation*	0.33	0.50	1	4
*LULC*	0.16	0.20	0.25	1

Matrix calculations were performed to determine the eigenvectors (*W*), calculate the vector product, and calculate the maximum lambda (eigenvalue). It was possible to determine the values of 0.04 for the Consistency Index (CI), 0.89 for the Random Index (RI), and 0.04 for the consistency ratio (CR), indicating that the weighting was performed appropriately, according to Saaty (2003).

**Fig. 8:**
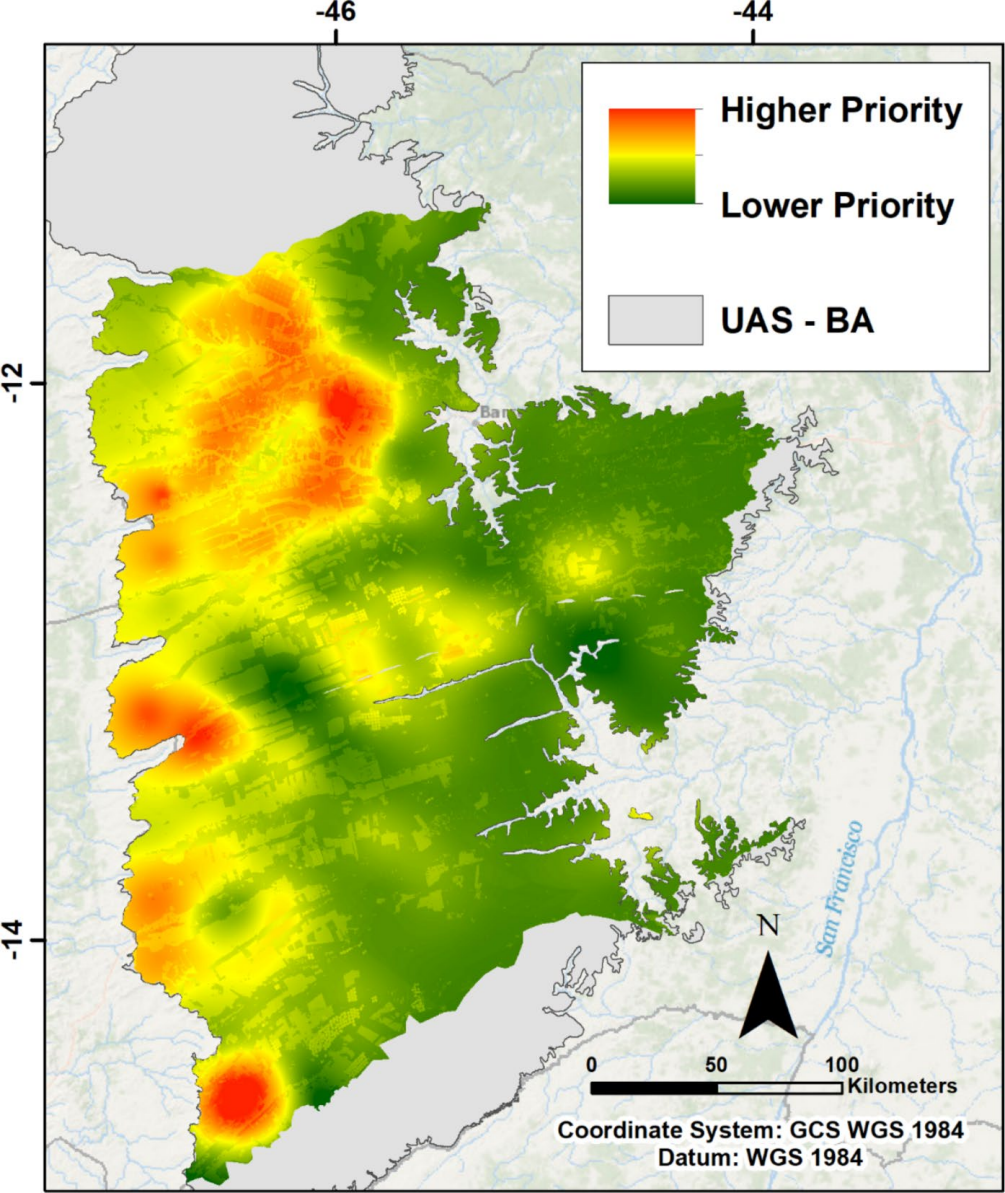
Map of the MCDM model with priority zones for the installation of new groundwater level monitoring points. Green areas indicate lower priority, and red areas indicate higher priority zones

Finally, Table 3 is generated with the relative weights for each criterion and the weighted parameters are overlaid in the GIS environment using the raster calculator, in which the normalized maps are multiplied by their respective weights and then overlaid, generating the final map of priority zones for monitoring (Fig. 8).

**Table 3:** Weight assigned to each criterion in the priority zones model

*Parameter*	*Assigned weight*
*Density*	0.43
*Decline rate*	0.30
*GWL variation*	0.21
*LULC*	0.06

The map generated by overlaying the weighted parameters indicates that the far western part of the study area, as well as the sub-basins of the Branco, Rio de Janeiro, de Ondas, and das Fêmeas rivers, should be prioritized for the installation of the complementary monitoring network. This is explained by the high density of interference points in these sub-basins, by the overlap of the variation factors and the rate of decline of GWL, which indicates greater drawdowns measured directly in the extreme west. The more pronounced expansion of agriculture, coupled with the concentration of pivots in these regions, also plays an important role.

### Correlation and definition of priority zones

To correlate the results obtained from the application of the different methodologies proposed, as relative to cross-validation, the eight wells with the higher errors in the estimated hydraulic head value and the four wells with the lower errors were selected. An attempt was made to identify whether there was any correlation with the RIMAS network wells’ GWL variation patterns, lithological characteristics of the well profiles, filter positioning, and spatial location in the study area.

**Fig. 9:**
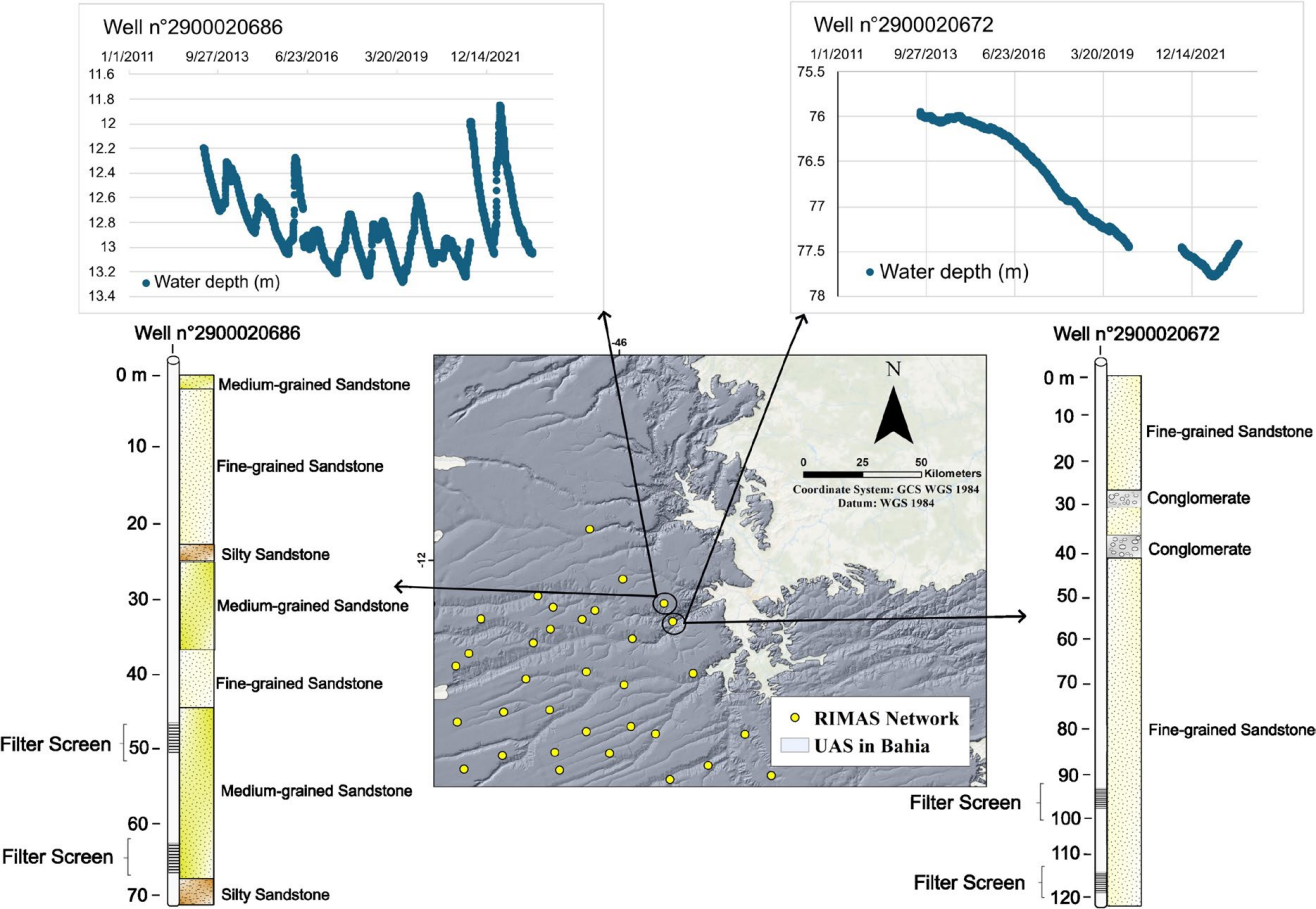
Map indicating the positions of wells no. 2900020686 and no. 2900020672 in the study area, showing that although close to each other, they exhibit different GWL variation patterns, with filter positioning indicating that they may be monitoring different aquifers locally

The wells with the higher associated error are usually isolated, with no other measured points nearby. However, as mentioned above, this is not the case for wells 2900029484 and 2900020686, which are in more densely populated areas. When associated with hydrogeological analysis, well 2900020686, which has the second higher measurement/estimation error (573/654 m), has a shallow aquifer GWL variation pattern, influenced by the climate (seasonality), with water level around 12.50 m, while the nearest well has the characteristics of a deeper aquifer with a water level around 77 m (Fig. 9). Similarly, well 2900029484, which has the fifth higher measurement/estimation error (734/689 m), has GWL varying between 26 and 31 m deep, while two of the three surrounding wells show a shallow aquifer pattern (GWL < 10 m), with seasonality influence, and the third shows a slight continuous drop in GWL. According to the lithological profile, this last well has a layer of silicified sandstone around 4 m thick, which may indicate it is monitoring another subtype of aquifer.

Regarding the four points with the lowest associated error, wells 2900024874 and 2900024882, with measurement/estimation errors of less than 0.2 m, are located in the most densely populated areas of the study area. Well 2900024874 has a GWL variation pattern indicating an aquifer influenced by seasonality, with the three closest points showing the same pattern. The same is true for wells 2900024882, 2900020685, and 2900024877, which showed smaller errors, sequentially.

Correlation of the hydrogeological analysis with the cross-validation during interpolation by ordinary kriging shows that, in general, the wells with the higher associated errors are isolated in outlying zones of the study area or have points around them with different patterns of GWL variation. On the other hand, the wells with the lower errors are in more densely monitored areas and tend to have surrounding wells with the same pattern of GWL variation. This may indicate that the positioning of the filters could influence the error due to the monitoring of different aquifer units. Although the geostatistical method only considers the mathematical relationships related to the interpolated variable (the hydraulic head), not taking into account qualitative aspects, such as the variation of the GWL, this classification is directly influenced by the positioning of the filters along the lithological profiles of the monitoring wells, as well as the hydraulic head values, thus validating the correlation made here.

The results obtained with the geostatistical method emphasize the need to expand the existing network. In comparison with other studies that have also applied interpolation by ordinary kriging, the standard error of the present work varies spatially by dozens of meters over the interpolated area, while Bhat et al. (2015) obtained error values ranging from 0 to 3.60 m for salinity-adjusted heads in an area in the southeastern USA; Theodossiu & Latinopoulos (2006) obtained errors ranging from less than 10 to more than 60 m in a basin in northern Greece, and Singh and Katpatal (2017) obtained values between 0.8 and 2.2 m in the Wainganga sub-basin in India. The studies cited were carried out in areas of much smaller and more densely populated extensions, with monitoring wells, ranging from one well every 13 km^2^ to one well every 580 km^2^, while the study area of the present work has one well every 1049 km^2^.

The application of the MCDM method, although the present study used direct measurement data of GWL variation in the monitoring wells, proved to be a good alternative for areas with limited direct measurement data, making it possible to use maps related to physiographic and anthropogenic use characteristics to define vulnerable areas. This corroborates the findings of other studies that used this methodology, either for the design of quality or level monitoring networks (Esquivel et al., 2015; Esquivel-Martínez et al., 2023; Patoni et al., 2024; Taheri et al., 2020).

Integrating the results obtained with the interpolation of the error and the MCMD model indicate a need to prioritize monitoring in the Branco and Rio de Janeiro sub-basins, in the northern region of the Upper Grande River basin. The geostatistical method also indicated the need for a better monitoring in the São Desidério River sub-basin, and in the downstream area of the Formoso River sub-basin (Fig. 6), unlike the MCDM model, which indicated greater priority along the entire western end of the study area and also in the Ondas and Fêmeas River sub-basins (Fig. 8). This is explained by the fact that interpolation by kriging of the standardized mean error considers the points where there are measured values, and consequently, the sub-basins mentioned will have greater errors because they have a lower number of monitoring wells. In the case of the multi-criteria analysis, parameters such as the density of abstraction points and direct measurements of drawdown in the RIMAS wells were considered. As a result, the zones defined as priorities are those with the greater measured drawdowns (extreme west) and the greater number of granted interference points (north of the Upper Grande River basin), while the sub-basins defined as priorities using the geostatistical method end up being less relevant here, as they have no monitoring points.

In the present study, we did not intend to assess the impact on the standard error in predicting the hydraulic head of the new monitoring points to be installed since, considering that the current network is still in its early stages, the distribution of the wells could be more widely spaced throughout the study area; however, priority was given to monitoring the areas that are most pressured and impacted by the demand for water resources, as demonstrated in the MCDM model combined with less populated areas according to kriging interpolation. Likewise, it was possible to assess with the geostatistical method that wells 2900024874 and 2900024882 are candidates to be removed from the network taking optimization into account, as they present redundancy, with error values of less than 0.2 m as described above. However, it is recommended that this should be carried out at a future stage, after expansion, when more effort will be needed to collect the data, considering the low density of monitoring points currently in place.

The methodologies applied and the expansion of the monitoring network in the UAS in the State of Bahia proposed here will enable subsidies to be provided for better management of water resources in the region, with a better understanding of the hydrodynamic aspects of the aquifer. In addition, the data generated could help answer questions that have been raised in recent studies about the impacts on groundwater and surface water, which are directly linked in UAS, together with attempts to answer whether the main protagonists in these changes are climatic factors, overexploitation, or both (Marques et al., 2020; Vieira, 2021; Leão et al., 2023; Mattiuzi, 2024). This is corroborated by studies such as Pousa et al. (2019) and Santos et al. (2020), which draw attention to the need to expand the hydrometeorological monitoring in western Bahia, especially at the sub-basin scale, citing two of the sub-basins proposed for monitoring in this study (Rio de Janeiro and Rio Branco rivers), which are reaching the threshold of water use for irrigation.

## Conclusions

The results of the present study, with the analysis of the current monitoring network (RIMAS) in one area where the density of monitoring wells is still very low, proved it difficult to estimate the variation in water levels regionally in the Bahia portion of the Urucuia Aquifer System. This hinders the effort to assess the impacts related to pumping and climate change. The RIMAS wells have simplified construction-lithologic profiles, making it hard to characterize the aquifer units and to determine which formation is being exploited. In addition, many wells have filter sections in more than one layer, which can lead to water mixing, both chemically and hydraulically. It is therefore recommended that the future complementary network to be implemented be installed in wells with well-known geological logs and restricted filter sections.

The application of geostatistics with ordinary kriging interpolation showed that the northern (Branco and Rio de Janeiro river sub-basins), eastern (São Desidério river sub-basin), and southern (Formoso river sub-basin) portions are the least densely monitored and have higher average standardized error, making them a priority for monitoring according to this methodology. Meanwhile, the MCDM model with superimposed parameters indicated that the extreme western region of the study area, as well as the sub-basins of the Ondas, das Fêmeas, Branco, and Rio de Janeiro rivers, are the priority zones for monitoring, considering the parameters used.

Thus, the combination of methodologies applied here indicated a greater need for monitoring in the sub-basins of the Rio Branco and Rio de Janeiro, but also along various sub-basins in the Upper Grande and Corrente River basins, with an emphasis on the western region of both watersheds. The proposal to expand monitoring presented here took into account the results obtained, while recognizing the need for a more uniform expansion of the network in the UAS, giving priority to the catchments most stressed in terms of water demand.

Considering the objective initially set out in this work, we demonstrated through the application of a hybrid approach to the design of monitoring networks that the application of geostatistics made it possible to analyze the existing network, showing the large errors associated with the lack of monitoring points, and the application of the MCDM method, taking into account aspects related to groundwater and surface water as well as aspects related to agriculture, the main activity in the region, and anthropogenic action, made it possible to delimit the priority zones for monitoring. On a secondary level, the hydrogeological analysis of the RIMAS wells failed to clearly delineate the subtypes of aquifers currently monitored by the network in the study area, due to the simplified lithological profiles and the multiple filter sections often scattered along the construction profiles.

We also recommend the expansion of the network to the Middle Grande and Carinhanha River basins in the future, considering the continued expansion of the agricultural frontier in MATOPIBA (acronym for Maranhão, Tocantins, Piauí and Bahia states’ region). Together with the expansion of the groundwater monitoring network, it is also important to expand the monitoring stations for fluviometric and meteorological data to improve data correlation and interpretation. The proposed network can also be used in the design and development of a quality monitoring network to measure and mitigate the possible impacts of agricultural expansion in the region, by monitoring parameters associated with the application of agrochemicals and pesticides. Finally, the application of remote sensing methods to groundwater monitoring networks in the future is encouraged, specifically in the UAS domain, considering its large extent and complex management taking into account physiographic, hydrogeological, and anthropic factors.

## Supplementary Information

Below is the link to the electronic supplementary material.

ESM1 (XLSX 14.1 MB)

ESM2 (XLSX 13.3 KB)

## Data Availability

No datasets were generated or analysed during the current study.
